# Clinical Features of Multiple Endocrine Neoplasia Type 4: Novel Pathogenic Variant and Review of Published Cases

**DOI:** 10.1210/jc.2019-00082

**Published:** 2019-04-16

**Authors:** Anja Frederiksen, Maria Rossing, Pernille Hermann, Charlotte Ejersted, Rajesh V Thakker, Morten Frost

**Affiliations:** 1 Department of Clinical Genetics, University of Southern Denmark, Odense, Denmark; 2 Department of Clinical Research, Faculty of Health, University of Southern Denmark, Odense, Denmark; 3 Center for Genomic Medicine, Rigshospitalet, Copenhagen University Hospital, Copenhagen, Denmark; 4 Department of Endocrinology, Odense University Hospital, Odense, Denmark; 5 Academic Endocrine Unit, Oxford Centre for Diabetes, Endocrinology & Metabolism, Radcliffe Department of Medicine, University of Oxford, Churchill Hospital, Oxford, United Kingdom

## Abstract

**Context:**

The clinical phenotype of multiple endocrine neoplasia type 4 (MEN4) is undefined due to a limited number of published cases. Knowledge on disease manifestation in MEN4 is essential for developing prevention programs and treatment.

**Objective:**

To expand current knowledge of the MEN4 phenotype including assessment of penetrance.

**Design:**

This is a case report and a brief review of previously published MEN4 cases.

**Patients:**

We report a large Danish family with multiple cases of endocrine tumors that segregated with a pathogenic variant in the *CDKN1B* gene.

**Main Outcome/Result:**

The medical history of the proband included primary hyperparathyroidism and Cushing disease. Genetic analysis identified a pathogenic variant in *CDKN1B* (c.121_122delTT, p.Leu41Asnfs*83). Among the family members, another 12 individuals were identified as carriers of the same variant, which segregated with development of endocrine tumors. Hypercalcemia due to primary hyperparathyroidism occurred in all 13 of the available carriers of the genetic variant, and 4 patients also had functioning or nonfunctioning pituitary adenomas, whereas 1 patient had a metastatic neuroendocrine tumor (carcinoid). Loss-of-heterozygosity was detected in two of five parathyroid adenomas, supporting that *CDKN1B* acts as a tumor suppressor gene. Thirty cases representing 16 different *CDKN1B* variants have previously been reported, and these cases presented primarily with primary hyperparathyroidism and functioning and nonfunctioning pituitary tumors.

**Conclusion:**

Hypercalcemia due to primary hyperparathyroidism and pituitary tumors are common in MEN4. Gastrointestinal neuroendocrine tumors appear to be less prevalent in MEN4 than in MEN1.

## Teaser

This study shows the clinical phenotype of a pathogenic variant that associates with MEN4 and provides an overview of previous MEN4 cases.

Multiple endocrine neoplasia (MEN) encompasses a group of diseases characterized by the existence of tumors in two or more endocrine organs in a patient (1). The occurrence of tumors within specific organs has given rise to distinct subtypes of MEN, including MEN1 to -4 (2, 3). Patients with MEN1 may develop parathyroid, pituitary, adrenocortical, gastroenteropancreatic neuroendocrine, and carcinoid tumors as well as lipomas, collagenomas, meningioma, and facial angiofibromas. The clinical presentation of MEN1 varies considerably as MEN1-related tumors may manifest due to local tumor growth, tumor burden, or metastases as well as hormonal hypersecretion, which can lead to development of specific clinical phenotypes associated with primary hyperparathyroidism, prolactinomas, acromegaly, Cushing disease, gastrinomas, and insulinomas (1). Likewise, MEN2 and MEN3 are characterized by development of endocrine tumors including primary hyperparathyroidism and pheochromocytoma. In addition, patients with MEN2 and -3 develop medullary thyroid carcinoid tumors, and the clinical presentation of MEN3 also includes mucosal neuromas (4).

MEN1 to -3 are autosomal-dominant diseases associated with mutations in the *MEN1* (MEN1) and *RET* (MEN2 and -3) genes, which act as tumor suppressor gene and an oncogene, respectively. Although patients with MEN2 or MEN3 carry a germline mutation in *RET*, mutations in *MEN1* are only identified in 85% to 90% of patients with MEN1 (1, 5). Germline mutations in the cyclin-dependent kinase (CDK) inhibitor 1b gene (*CDKN1B*) were identified in some patients that presented with a MEN1 phenotype but did not have a *MEN1* mutation (6, 7), and these cases have been classified as MEN4 (3, 8). It has been estimated that 3% of patients with a MEN1-like phenotype without a mutation in *MEN1* carry a mutation in *CDKN1B* (3), but mutations in other *CDKN* genes have also been reported in patients with a MEN1-like phenotype (7). MEN1 encodes menin, which is involved in cell division, genome stability, and gene transcription (3), whereas *CDKN1B* encodes p27, which prevents cell cycle progression and acts as a tumor suppressor gene (9). Moreover, menin, which promotes histone methylation, affects gene transcription of cell cycle regulators including p27 (10, 11).

However, with only 29 cases with MEN4 including 16 different mutations reported, it remains undetermined if the clinical phenotypes of MEN1 and MEN4 are similar. Parathyroid tumors, which lead to primary hyperparathyroidism, are the most consistently reported type of tumors in patients with MEN4, but neuroendocrine tumors including pituitary, adrenal, and enteropancreatic tumors as well as tumors involving nonendocrine organs, such as lipomas and meningiomas, have been observed in some patients with MEN4 (6–8, 12–15). However, it remains to be established if there is a direct association between genotype and phenotype in patients with MEN4, and the expressivity and penetrance of the disease is only partly described. Reports on segregation of the MEN4 phenotype with a mutation in *CDKN1B* in multiple generations are very limited. Pellegata *et al.* (6) reported a pathogenic *CDKN1B* variant in two sisters presenting a MEN1-like phenotype. Haplotype analysis showed that the mutated allele was inherited from the father, who was diagnosed with acromegaly, supporting that MEN4 is inherited in an autosomal-dominant manner.

Characterization of the individual features of MEN4-related disease manifestation is important for patient management, and the paucity of reports on cases with MEN4 impedes the development of screening and treatment guidelines. This report describes a large family with several members with a MEN1-like phenotype that included primary hyperparathyroidism associated hypercalcemia and different types of endocrine tumors. Genetic investigations revealed a pathogenic variant in *CDKN1B* in 13 family members, which segregated with the disease in two generations, supporting that MEN4 is an autosomal-dominant disorder.

## Materials and Methods

### Index case story

**Figure 1.**
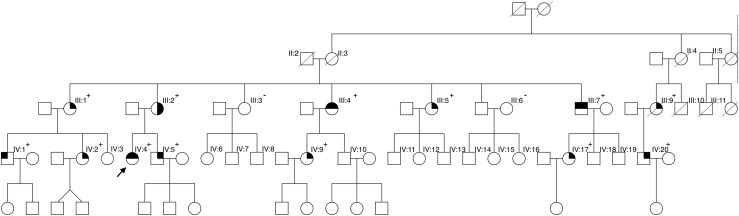
Pedigree showing a family with 13 *CDKN1B* mutation carriers. Pituitary adenoma is indicated by a circle with the top left fourth shaded; hyperparathyroidism by a circle with the top right fourth shaded; tumor unspecified by a circle with the bottom left fourth shaded; neuroendocrine tumor by a circle with the bottom right fourth shaded; male is indicated by an open square; female is indicated by an open circle; and deceased is indicated by a line through the symbol. +, mutation positive; −, mutation negative.

The proband, a 38-year-old white woman (Fig. 1, IV:4), was referred for an endocrine assessment due to development of asymptomatic hypercalcemia.

#### Medical history

The patient had been diagnosed with polycystic ovarian syndrome (PCOS) due to amenorrhea, hirsutism, polycystic ovaries, and elevated levels of testosterone at 30 years of age. Aged 36, she had been reassessed due to increasing body weight, hair loss, as well as persisting amenorrhea and hirsutism. The clinical assessment revealed increased levels of testosterone (3.83 mmol/L), normal adrenal morphology based on a CT scan as well as normal corticotrophin (14 pmol/L), and 24-hour urinary cortisol levels (45 nmol/d). The symptoms remained despite weight loss and metformin treatment. Also, mild hyperparathyroid hypercalcemia was observed and further investigated.

#### Clinical presentation and biochemical tests at assessment of hypercalcemia

The patient did not report fatigue or symptoms related to depression, there were no gastrointestinal symptoms, thirst or frequent urination, bone pain, or muscle weakness at time of assessment due to hypercalcemia, and there was no history of renal stones or fractures. There was no use of medications known to influence bone or calcium metabolism.

Biochemical tests revealed mildly elevated ionized calcium levels (1.32 to 1.40 mmol/L) and nonsuppressed PTH levels (2.4 to 4.3 pmol/L). The level of phosphate was normal (0.80 to 0.96 mmol/L), and serum levels of 25-hydroxy vitamin D and 1.25-hydroxy vitamin D were within the normal ranges (61 to 90 nmol/L and 108 pmol/L, respectively). Furthermore, the urinary calcium-creatinine ratio was 0.011, and DXA showed normal bone mineral density at the lumbar spine and femoral neck. These investigations suggested that the patient had primary hyperparathyroidism, but the low calcium-creatinine ratio did not exclude familial hypocalciuric hypercalcemia. As the patient was only 37 years old, she was referred for genetic testing of monogenetic causes of primary hyperparathyroidism, as recommended by the current guidelines (16), which revealed a pathogenic variant in *CDKN1B*, and the patient was subsequently diagnosed with MEN4.

#### Clinical presentation after diagnosis of MEN4

Subsequent measurement of plasma vasointestinal peptide, gastrin, pancreatic polypeptide, 5-hydroxyindoleacetic acid, and metanephrines were normal. However, aged 37 years, the patient reported hot flushes, and the clinical examination now exposed lipodystrophy at the neck, red striae on the abdomen, and central adiposity. Urinary free cortisol and corticotrophin levels were increased (559 nmol/d and 22 pmol/L, respectively), endogenous cortisol secretion was not suppressed on a 1-mg dexamethasone suppression test, and MRI revealed the presence of a pituitary tumor (6 mm in diameter), consistent with Cushing disease. Transphenoidal resection of the tumor was performed, with complete removal of the tumor that resulted in normalization of cortisol levels. Histological and immunohistochemical investigations demonstrated well-differentiated cells resembling corticotrophs with intense staining for ACTH, confirming the clinical diagnosis of Cushing disease. Postoperative levels of ACTH, 24-hour free cortisol, as well as TSH and T4 levels were normal.

### Genetic screening

Genomic DNA was purified from whole blood using the QIAamp DNA Mini Kit (Qiagen, Hilden, Germany) or ReliaPrep Large Volume HT gDNA Isolation Kit (Promega, Madison, WI) using a Tecan Freedom EVO HSM2.0 Workstation (Promega) according to the manufacturer’s instructions. Screening for genetic variants in *MEN1* (NM_130799), *CDC73* (NM_024529), *CASR* (NM_000388), *RET* (NM_020975), and *CDKN1B* (NM_004064) were done by targeted next-generation sequencing and a library designed to capture all exons from the genes above as previously described (16). Sequencing was performed on a MiSeq (Illumina, San Diego, CA) to an average depth of at least ×100. Sequencing data were analyzed using Sequence Pilot (JSI Medical Systems, Ettenheim, Germany), in which variants were called if the nonreference base frequency was >25%. Sanger sequencing verified sequence variants in an independent blood sample.

### Loss-of-heterozygosity analysis

To access possible loss-of-heterozygosity (LOH) of *CDKN1B* in tumor tissue from the index case and her family members, DNA was extracted from formalin-fixed paraffin-embedded tissue of tumor samples and analyzed with OncoScan array (Affymetrix, Santa Clara, CA) according to the manufacturer’s instructions. Raw data (.CEL files) were processed and analyzed using NEXUS (BioDiscovery, El Segundo, CA).

All participants provided informed and written consent for participation.

## Results

### Genetic investigations

#### Genetic screening of index case

Results of genetic screening (Fig. 1, IV:4) of *CaSR*, *RET*, intron-exon junctions, and coding regions as well as large deletions of *MEN1* and *CDKN1B* of (IV:4) revealed a pathogenic frame-shift variant in the *CDKN1B* gene (c.121_122delTT, p.Leu41Asnfs*83), which was classified as pathogenic according to the American College of Medical Genetics guidelines (17). Based on biochemical tests consistent with primary hyperparathyroidism, the history of Cushing disease, and genetic screenings, the patient was diagnosed with MEN4.

#### Genetic tests of family members

Family members were referred for genetic counseling. Fifteen subjects from two generations accepted predictive genetic testing, which identified a total of 13 carriers of the pathogenic variant (Fig. 1). Individuals carrying the variant were subsequently offered clinical assessment, including biochemical screening and MRI or CT scans of the pituitary gland and pancreas.

### Family history of family members

#### Primary hyperparathyroidism

All carriers of the *CDKN1B* variant with available measurements of calcium and PTH levels had mild, asymptomatic hypercalcemia due to primary hyperparathyroidism (measurements were not available in one case), with levels of ionized calcium concentrations ranging from 1.33 to 1.50 nmol/L and PTH concentrations between 2.5 and 12.0 pmol/L (Table 1). None of the carriers reported renal stones or skeletal fractures. One case (III:9) reported of a subtotal parathyroidectomy due to symptomatic primary hyperparathyroidism performed 9 years prior to the clinical assessment. Although three of her parathyroid glands had been removed, increased levels of calcium and parathyroid hormone were observed during the follow-up of the family, indicating recurrence or development of primary hyperparathyroidism in the remaining parathyroid gland.

**Table 1.** Clinical Presentation of Cases of MEN4 Belonging to a Danish Family, Including Measurements of Ionized Calcium and PTH, Diagnosis of Primary Hyperparathyroidism, MRI Scans of the Pituitary Gland, Abdominal CT, or MRI Scans and Additional Scans and Medical History

Subject Identification Number (Sex)	plasma*p*-Ca^2+^ (mmol/L)	*p*-PTH (nmol/L)	PHPT	Pituitary Tumor (Size of Tumor) (Age at Debut)	Abdominal Scans	Additional Medical History of Cancer or Endocrine Disorders
III:1 (F)	1.44	8.1	PHPT	No tumors	CT abdomen: no tumors	CT thorax: benign lung tumor (12 mm)
III:2 (F)	NA	NA	PHPT	No tumors	CT abdomen, PET-CT, and EUS: carcinoid tumor in pancreas (67 y)	CT thorax: no tumors; hypothyroidism
III:4 (F)	1.38	6.4	PHPT	Nonfunctioning microadenoma (6.7 × 3.9 mm) (66 y)	CT abdomen: no tumors	CT thorax: no tumors
III:5 (F)	1.35	2.8	PHPT	No tumors	CT abdomen: no tumors	Goiter
III:7 (M)	1.33	4.8	PHPT	Nonfunctioning microadenoma (4.5 × 3 mm) (64 y)	CT abdomen: no tumors	CT thorax: no tumors
III:9 (F)	1.50	12	PHPT	No tumors	NA	
IV:1 (M)	1.33	4.9	PHPT	Nonfunctioning, asymptomatic macroadenoma (14.8 × 10.5 mm) (46 y)	CT abdomen: no tumors	CT thorax: no tumors
IV:2 (F)	1.36	6.4	PHPT	No tumors	CT abdomen: no tumors	Hirsutism
IV:4 (F)	1.37	2.5	PHPT	Microadenoma (ACTH-producing) (6 mm in diameter)	NA	PCOS
IV:5 (M)	1.33	3.2	PHPT	No tumors	MR abdomen: no tumors	
IV:9 (F)	1.33	5.1	PHPT	No tumors	MR abdomen: no tumors	PCOS
IV:17 (F)	1.33	6.7	PHPT	No tumors	NA	
IV:20 (M)	1.37	5.2	PHPT	No tumors	CT abdomen: no tumors; pancreatitis sequelae	CT thorax: no tumors

Abbreviations: EUS, endoscopic ultrasound; F, female; M, male; NA, not available; PET, positron emission tomography; p-Ca++, plasma ionized calcium; p-PTH, plasma parathyroid hormone; PHPT, primary hyperparathyroidism.

#### Pituitary tumors

Besides the proband (IV:4), nonfunctioning pituitary tumors were identified in three carriers of the *CDKN1B* variant, including two microadenomas (III:4 and III:7) and one macroadenoma (IV:1) (Table 1), with functionality classified on the basis of measurements of IGF-1 and prolactin.

#### Other neuroendocrine tumors

The mother (III:4) of the proband was diagnosed with metastatic neuroendocrine tumor (carcinoid) and treated with a somatostatin analog (lanreotide). Immunohistochemical assessments showed expression of the intestine-specific transcriptional factor CD2X, but neither CT nor MRI identified the site of the primary tumor.

#### Other disorders

Two of the family members were known to have goiter, and one individual was diagnosed with hypothyroidism during the follow-up of the family. In addition, three of the family members reported that they had PCOS and/or hirsutism. Furthermore, one case (III:9) died unexpectedly aged 77 years, and the postmortem inspection revealed a 5 × 5 cm ulcer on the right breast, indicating that the patient had breast cancer.

### LOH

Tissue samples from five tumors including three parathyroid tumors, one pituitary tumor, and a metastasis from a carcinoid tumor were retrieved from surgical specimens stored at public hospitals. Genetic tests of these biopsies showed LOH in two out of five biopsies, including two cases of parathyroid adenomas but not in the pituitary tumor of the proband (Table 2).

**Table 2.** Investigations of LOH in Tissue Samples From MEN4 Cases

Patient Identification Number	Tissue	*CDKN1B*_CNV Results	NGS_reads of *CDKN1B* Variant c.121_122delTT, %
III:9	Parathyroid adenoma	Normal	46
IV:4	Pituitary adenoma	Normal	49
III:1	Parathyroid adenoma	LOH	72
III:2	Parathyroid adenoma	LOH	79
III:2	Carcinoid tumor (metastasis)^*a*^	Normal	60

^a^ Tissue was scarce, and sample included only 40–50% tumor tissue.

### Previous reports on MEN4

Previous reports describing the clinical phenotype of MEN4 comprise a total of 29 *CDKN1B* mutation-positive individuals and include 16 different mutations (Table 3) (18–24). The most commonly reported MEN4-related disorder was primary hyperparathyroidism, with 11 cases of the disease in 16 carriers of a mutation. Currently available reports of the index cases also include six cases of gastroenteropancreatic neuroendocrine tumors, including two cases of gastrinomas (in five patients) as well as five cases of pituitary tumors, comprising four cases of acromegaly and one case of Cushing disease. Colon cancer has been reported in one family with a *CDKN1B* mutation (20), but manifestation of endocrine disease was unreported, and *in silico* evaluation or functional studies of the mutation were not described.

**Table 3.** Previously Reported Cases With *CDKN1B* Mutations and Associated Clinical Phenotypes

First Author, Reference	Genotype	Proband, Male/Female (Age at Diagnosis)	MEN4 Phenotype (Age at Diagnosis of Disease)	Endocrine Histology	Mutation-Positive Cases Reported	Family History	LOH in Tumor
Missense mutation							
Costa-Guda *et al.* (18)	c.25G>A (p.G9R)	M (68 y)	PHPT (68 y)	Parathyroid adenoma	n = 1	No	No
Belar *et al.* (19)	c.163G>A, (p.A55T)	F (42 y)	Zollinger-Ellison syndrome (42 y)	NA	n = 1	None	NA
			PHPT (51 y)				
Esteban-Jurado *et al.* (20)	c.195G>T, (p.Q65H)	M (47 y)	Colon cancer (47 y)	NA	n = 4	Colon cancer in three generations	Yes
Molatore *et al.* (13)	c.206C>G, (p.P69L)	F (79 y)	Bronchial carcinoid tumor (67 y)	Parathyroid adenoma	n = 1	NA	No
			PHPT (67 y)				
			Nonfunctioning pituitary microadenoma (79 y)				
			Papillary thyroid carcinoma (64 y)				
Pellegata *et al.* (6)	c.227G>A (p.W76X)	F (30 y)	Acromegaly (30 y)	Invasive pituitary adenoma (growth hormone secreting)	n = 4	Father had acromegaly, and sister had renal angiomyolipoma (55 y)	
			PHPT (46 y)				No (angiomyolipoma)
Agarwal *et al.* (7)	c.283C>T, (p.P95S)	F (50 y)	Zollinger-Ellison syndrome (50 y)		n = 1		NA
			PHPT (3 parathyroid tumors)				
			Masses in duodenum and pancreas (50 y)				
Tichomorowa *et al.* (21)	c.286A>C, (p.P96Q)	F (NA)	Acromegaly		n = 1	Maternal aunt with acromegaly	NA
	c.356T>C, (p.I119T)	F (NA)	Acromegaly	NA	n = 1	Maternal aunt with acromegaly	NA
Elston *et al.* (22)	c.378G>C, (p.E126D)	F (15 y)	Nephrocalcinosis (15 y)	Parathyroid adenoma	n = 3	Mother (46 y)	NA
			PHPT			Maternal grandfather (74 y)	
						Both mutation positive	
						No symptoms	
Costa-Guda *et al.* (18) and Bugalho *et al.* (23)	c.397C>A, (p.P133T)	F (53 y)	Parathyroid adenoma (53 y)	Parathyroid adenoma	n = 1	No	No
			Parathyroid adenoma (56 y)	Papillary thyroid carcinoma			
Bugalho *et al.* (23)		F (56 y)	Papillary thyroid carcinoma (56 y)	Parathyroid adenoma	n = 1		
			Cerebral meningioma (NA)				
Agarwal *et al.* (7)	ATG-7G>C	F (50 y)	PHPT (50 y)	Parathyroid adenoma	n = 3	Two mutation-positive but asymptomatic daughters	No
Agarwal *et al.* (7)	c.595T>C, (p.*199Q)	F (NA)	PHPT (NA)		n = 3	Monozygotic twin with PHPT	NA
						Maternal aunt and cousin with PHPT	
Small deletions							
Occhi *et al.* (8)	c.-456_-453delCCTT in 5′ UTR	F (62 y)	Acromegaly (62 y)	Pancreatic tumor	n = 1	NA	No
			Endocrine neoplasm pancreas, nonfunctional (62 y)				
Malanga *et al.* (14)	c.-32_-29delGAGA in the 5′ UTR	F (69 y)	Gastric carcinoid tumor (69 y)		n = 1	None	NA
			PHPT (74 y)				
Tonelli *et al.* (12) and Pardi *et al.* (24)	c.374_375delCT (p.S125X)	F (41 y)	Hashimoto thyroiditis (41 y)	Parathyroid adenoma	n = 2	Son, mutation positive without symptoms	LOH
			PHPT, two parathyroid adenomas (41 y)				
			Pancreatic neuroendocrine tumor (Zollinger-Ellison) (51 y)				
			Neuroendocrine neoplasia in duodenal walls (52 y)				
Duplication							
Georgitsi *et al.* (15)	c.59_77dup19	F (45 y)	Neuroendocrine cervical carcinoma (45 y)	Neuroendocrine cervical carcinoma	n = 1	None	Yes
			Cushing disease (46 y)				
			PHPT (47 y)				
			Multiple sclerosis (46 y)				

Abbreviations: F, female; M, male; NA, not available; UTR, untranslated region.

In addition, sporadic cases of bronchial carcinoid, meningioma, cervical neuroendocrine carcinoma, and papillary thyroid carcinoma but not thymic carcinoma have been reported in patients with MEN4 (Table 3).

The limited number of cases with MEN4 exclude appraisal of an association between genotype and phenotype, including the age of onset for tumor development. Primary hyperparathyroidism, the most commonly reported MEN4-associated disease, has been reported with onset as early as 15 years of age (22) but is generally diagnosed in individuals aged at least 40 years. Previous reports include one case of a pituitary tumor in a 30-year-old individual and gastrointestinal tumors diagnosed as early as 40 years of age. Furthermore, all previous adult *CDKN1B* mutation carriers had MEN4-related disease, indicating a complete penetrance.

## Discussion

In this study, we report a large family with several individuals characterized by development of primary hyperparathyroidism and other endocrine tumors, and these features of MEN segregated with a pathogenic variant in *CDKN1B* over two generations. To the best of our knowledge, this is the largest family of patients with MEN4 reported to date. With 30 previously reported carriers of presumed pathogenic *CDKN1B* variants, our 13 cases in the present report expand our knowledge of the clinical presentation of MEN4 considerably.

The present and previous reports on the clinical presentation of MEN4 support that hypercalcemia due to primary hyperparathyroidism develops in the majority of, if not all, patients with MEN4 (Tables 1 and 3) (7, 25). This is in accordance with the clinical presentation of MEN1, which is associated with an estimated penetrance of primary hyperparathyroidism of 90% (1). Primary hyperparathyroidism appears to be indolent in patients with MEN4, as mild hypercalcemia was observed in most of the cases. However, one case was hypercalcemic after subtotal parathyroidectomy, and recurrent primary hyperparathyroidism has previously been reported (12). The lowest age at diagnosis of primary hyperparathyroidism in the family reported in this study was 29 years (IV:17), but this specific manifestation of MEN4 has been diagnosed in a 15-year-old individual in a previous case report (22). However, information regarding the age of development of MEN4-related tumors is limited. Based on known cases, guidelines advocate that screening for primary hyperparathyroidism in patients with MEN1 is initiated at the age of 8 years. Pending further publication of clinical reports on the development of disease manifestations in patients with MEN4, it appears prudent to initiate screening of calcium and PTH levels in patients with MEN4 from the age of 15 years.

The prevalence of pituitary tumors in patients with MEN1 is ∼40%. In the Dutch MEN1 cohort, approximately half of the pituitary tumors including ∼50% nonfunctioning microadenomas were identified during screening (26). Assessment of the distribution of size and functionality of pituitary tumors in patients with MEN4 is challenging due to the low number of known cases. Reviewing previous MEN4 cases identified three cases of acromegaly and two cases of Cushing disease equaling tumor penetrance of ∼10% and 5%, respectively, which corresponds to the prevalence of somatotropinomas and corticotropinomas in patients with MEN1 (1). Whereas 20% of the pituitary adenomas in patients with MEN1 are classified as prolactinomas (1), this type of tumor has not been reported in patients with MEN4, possibly indicating differential effects of *CDKN1B* and *MEN1* mutations on development of lactotropic tumors in the pituitary gland. Future studies may confirm if these differences are genuine or arise due to the limited number of MEN4 cases. There are currently no cases of pituitary tumors in young individuals with MEN4, suggesting that screening for pituitary tumor development with measurements of prolactin and IGF-1 and MRI may begin in adolescents and not in children as suggested in patients with MEN1 (1). Pituitary tumors including nonfunctioning microadenomas in particular are common in the general population, and it is not possible to reliably distinguish clinically between MEN-related pituitary tumors and incidentalomas. In addition, the growth potential of nonfunctioning pituitary microadenomas in MEN4 is not known, although it is important to note that the growth potential of nonfunctioning pituitary microadenomas in patients with MEN1 is reported to be limited (26). Thus, it is difficult to provide guidance for the specific clinical management of nonfunctioning pituitary microadenomas in patients with MEN4, and it is suggested that clinical management should concur with the international guidelines for pituitary incidentalomas (27).

Our review of known MEN4 cases indicates that the prevalence of gastroenteropancreatic neuroendocrine tumors is lower in patients with MEN4 than in MEN1, in which 30% to 70% develop enteropancreatic tumors, and 10% develop gastric tumors (1). Gastrinomas and nonfunctional pancreatic tumors have been documented in previous MEN4 cases with a prevalence of ∼25%. The discrepancy in the observed penetrance of gastroenteropancreatic tumors may be explained by the fact that our investigation includes several mutation carriers identified as part of an assessment of the family history, whereas previous reports mainly rely on single cases who were identified due to specific symptoms or conditions including gastroenteropancreatic neuroendocrine tumors. Despite substantial improvement of the overall survival of patients with MEN1, gastroenteropancreatic neuroendocrine tumors, malignant nonfunctioning pancreatic neuroendocrine tumors in particular (25, 28, 29), remain associated with increased morbidity and mortality. Awaiting more information about the age of disease development including gastrointestinal neuroendocrine tumors, it appears prudent to screen for manifestations of MEN4 with biochemical tests and imaging (*e.g.*, MRI, CT, or endoscopic ultrasound), as recommended in guidelines for evaluation of patients with MEN1 (1).

Thus, clinical reports demonstrate similar phenotypes in patients with MEN1 and MEN4 despite differences in the molecular genetic background. The *CDKN1B* gene encodes a CDK inhibitor known as p27Kip1, which inhibits the cell cycle by preventing the transition from the G_1_ to the S phase through inhibition of CDK activity (30, 31). Although *CDKN1B* appears to inhibit the development of tumors, genetic investigations of MEN4-related tumors are limited in numbers and not consistently supporting that *CDKN1B* is a tumor suppressor gene. LOH and p27 deficiency was observed in a bronchial carcinoid and cervical neuroendocrine carcinoma from patients with MEN4, supporting that *CDKN1B* may act as a tumor suppressor gene; however, LOH was not observed in a parathyroid adenoma from one of these cases, and reduced expression of p27 was observed in a LOH-negative tumor, indicating that *CDKN1B* haploinsufficiency may lead to tumor development (13, 15). Additionally, loss of p27 was identified in a MEN4-related parathyroid adenoma despite no allelic loss (24). In accordance with previous publications, we observed LOH in a minority of the samples available for investigation, further substantiating that factors besides that of the *CDKN1B* mutation may be involved in tumor development in patients with MEN4. It has been suggested that LOH in MEN4-related tumors could be associated with a particularly aggressive neoplasia (12), but this is not supported by our cases that revealed LOH in two cases of asymptomatic primary hyperparathyroidism and no LOH in a metastasis from a carcinoid tumor. Although >90% of MEN1-related tumors exhibit LOH, currently available data indicate that other factors are likely to contribute to the development of tumors in patients with MEN4. It has been suggested that factors such as epigenetic alterations may explain the development and growth of tumors without LOH (5, 32). Furthermore, reduced expression of p27 may arise due to posttranslational ubiquitin-proteasome degradation (7, 33), although lower levels of p27 may be observed in cases of *CDKN1B* LOH or *CDKN1B* somatic mutations (13). Regrettably, as immunohistochemical tests were not included in this case report, we were unable to account for the effects on p27 expression in tumors.

## Conclusion

Currently available clinical data demonstrate that the main features of MEN4 are primary hyperparathyroidism as well as functional and nonfunctional pituitary and gastroenteropancreatic neuroendocrine tumors. As in MEN1, other types of tumors may develop in patients with MEN4, including carcinoid tumors and meningioma. Onset of disease appears to be later in patients with MEN1 than in those with MEN4, and development of a prolactinoma is common in MEN1 but rare in MEN4. Future studies that include larger numbers of patients with MEN4 will substantiate if disease manifestations vary between MEN1 and MEN4, and this will help the development of guidelines for evaluation and treatment of patients with MEN4.

## Abbreviations:

CDK: cyclin-dependent kinase
LOH: loss-of-heterozygosity
MEN: multiple endocrine neoplasia
PCOS: polycystic ovarian syndrome
